# How can we modulate aging through nutrition and physical exercise? An epigenetic approach

**DOI:** 10.18632/aging.204668

**Published:** 2023-04-20

**Authors:** Ana Teresa Rajado, Nádia Silva, Filipa Esteves, David Brito, Alexandra Binnie, Inês M. Araújo, Clévio Nóbrega, José Bragança, Pedro Castelo-Branco, ALFA Score Consortium

**Affiliations:** 1 Algarve Biomedical Center, Research Institute (ABC-RI), University of Algarve Campus Gambelas, Faro 8005-139, Portugal; 2 Algarve Biomedical Center (ABC), University of Algarve Campus Gambelas, Faro 8005-139, Portugal; 3 Faculty of Medicine and Biomedical Sciences (FMCB), University of Algarve Campus Gambelas, Faro 8005-139, Portugal; 4 Department of Critical Care, William Osler Health System, Etobicoke, Ontario, Canada; 5 Champalimaud Research Program, Champalimaud Centre for the Unknown, Lisbon, Portugal

**Keywords:** epigenetics, aging, nutrition, caloric restriction, physical exercise

## Abstract

The World Health Organization predicts that by 2050, 2.1 billion people worldwide will be over 60 years old, a drastic increase from only 1 billion in 2019. Considering these numbers, strategies to ensure an extended “healthspan” or healthy longevity are urgently needed.

The present study approaches the promotion of healthspan from an epigenetic perspective. Epigenetic phenomena are modifiable in response to an individual’s environmental exposures, and therefore link an individual’s environment to their gene expression pattern. Epigenetic studies demonstrate that aging is associated with decondensation of the chromatin, leading to an altered heterochromatin structure, which promotes the accumulation of errors.

In this review, we describe how aging impacts epigenetics and how nutrition and physical exercise can positively impact the aging process, from an epigenetic point of view. Canonical histones are replaced by histone variants, concomitant with an increase in histone post-translational modifications. A slight increase in DNA methylation at promoters has been observed, which represses transcription of previously active genes, in parallel with global genome hypomethylation. Aging is also associated with deregulation of gene expression - usually provided by non-coding RNAs - leading to both the repression of previously transcribed genes and to the transcription of previously repressed genes.

Age-associated epigenetic events are less common in individuals with a healthy lifestyle, including balanced nutrition, caloric restriction and physical exercise. Healthy aging is associated with more tightly condensed chromatin, fewer PTMs and greater regulation by ncRNAs.

## INTRODUCTION

Aging is a complex time-dependent multifactorial biological process, involving a gradual decline in cognitive and physiological functions over time. This results in a reduced capacity to respond to stressors, which in turn increases morbidity and mortality [1–3]. Pathological phenotypes associated with aging include frailty (a condition associated with progressive physical and mental decline [4], chronic medical conditions, such as diabetes and cardiovascular disease [5, 6], visual impairment, for example age-related macular disease [7, 8], cancer [9], and neurodegenerative disorders, such as Alzheimer’s, Parkinson’s and Huntington’s diseases [10, 11], to name some of the most prevalent.

Nonetheless, and despite many studies on aging, this subject remains poorly understood [2, 12]. Over the last 7 decades, human lifespan has steeply increased, which was reflected in the global population – as a result, the population of individuals over age 60 has increased dramatically from 205 million in 1950 to 1 billion in 2019. According to a World Health Organization (WHO) prediction, by 2030, individuals over 60 years old will outnumber children younger than 10 years old, and by 2050 the over-60 population will number 2.1 billion [13]. On the one hand, this extended lifespan is a tribute to the technological advances achieved in the last century, in terms of health, medicine, sanitation, and education. On the other hand, such increase of aged populations sets pressure on societies to develop specialized policies and services for the elderly, to reduce the impact of this trend on our communities [13].

The decade of 2020–2030 was termed by the WHO as the “Decade of Healthy Aging”, with the target of sustainably extending healthspan [14]. WHO has defined “Healthy Aging” as the possibility for everyone to be and do what they value throughout their life. For older adults, this means remaining independent and capable of participating in their daily activities, even if affected by illness [15].

Strategies to improve healthy aging include lifestyle modification (to limit the effects of risk behaviors, namely tobacco consumption, and alcohol abuse), regenerative medicine and tissue/organ engineering, manipulation of genes and pathways associated with longevity, and pharmacological compounds to extend healthy lifespan [12, 16].

Epigenetic alterations and genomic instability are also potential targets for healthy aging interventions [17–19]. The importance of epigenetic alterations for healthy aging was suggested in 1995 by Herskind and colleagues, who reported on a large cohort of Danish twins, demonstrating that 25% of their longevity (26% for males and 23% for females) was related to DNA sequence [20]. The 75% unaccounted for was attributed to the modulation of age-associated genetic factors by non-heritable environmental influences such as diet habits, physical activity, tobacco consumption, as well as social interactions established and education [21, 22]. Notwithstanding, several studies were performed in both animal models and humans, that highlighted the relevance of particular genes such as insulin or insulin-like growth factor 1 (IGF-1), Forkhead box O 3 (FOXO3) and AMP-activated protein kinase (AMPK) [23, 24]. Also, these genes were shown to be drug targetable, therefore promoting healthspan [24].

During the last decades, several studies suggested a key role for epigenetics during the aging process, in contrast to the first hypotheses formulated, which attributed aging to the accumulation of mutations in the genome [25]. Recent studies developed by David Sinclair’s Lab further explored this topic, demonstrating that using a system that induces changes of the epigenome in mice accelerated approximately 50% the DNA methylation epigenetic clock [25, 26].

The present manuscript provides an overview of aging from the (epi)genetic perspective, and summarizes lifestyle strategies that can be adopted to potentiate healthier epigenetic modifications and consequentially slow down biological aging.

## Epigenetics

**Figure 1:**
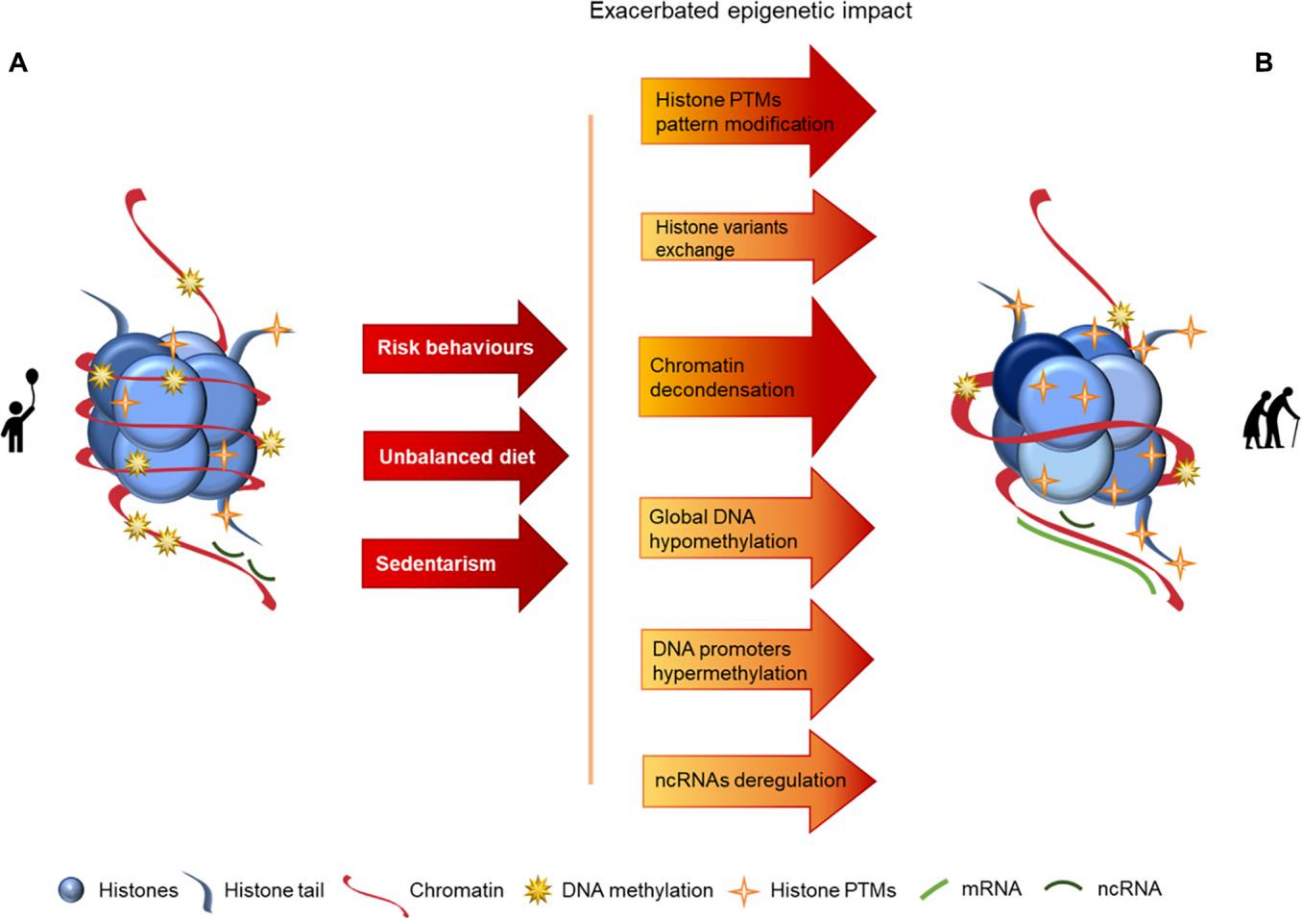
**Representation of age-associated epigenetic changes.** (**A**) Representation of a young individual chromatin: tight chromatin compactation, high levels of DNA methylation, few histone PTMs (particularly acetylation), canonical histones and balanced non-coding RNA regulation; (**B**) Representation of an old aged individual chromatin, we may observe a looser chromatin structure, lower levels of DNA methylation, higher levels of histones PTMs (particularly acetylation), histone variants presence, chromatin remodeling and disturbances in ncRNA regulation, what is reflected in an overall chromatin instability increase, when compared with the structure presented in A. The transition between the structures presented in A and B is represented by arrows. The arrows in red present the causes (here presented as a lifetime of risk behaviors (namely tobacco and alcohol consumption), unbalanced diet and sedentarism) whose effects are reflected by the arrows in orange/red, where darker colors represent more pronounced epigenetic alterations.

Epigenetics can be defined as *de novo* or inherited reversible modifications of the genome, which can affect gene expression without altering the DNA sequence. Epigenetic alterations are mediated by multiple mechanisms, of which histone modifications, DNA methylation and changes in non-coding RNAs expression are the best studied [18, 27] and whose alterations with aging are schematized in Figure 1.

Eukaryotic DNA is organized into higher-order chromatin through nucleosomes, which are core particles composed of histone octamers. Each octamer is constituted by two of each core histone proteins (H2, H3A, H3B and H4), around which 147 base pairs of DNA are wrapped [28, 29]. The linker histone H1 is also found in proximity to this core, promoting its stability [30]. Nucleosome core particles are arranged on the DNA like “beads on a string”, with a distance of approximately 200 base pairs between “beads”. This open DNA conformation is termed “euchromatin”. Additionally, the tightly packed or closed conformation that DNA and nucleosome core particles adopt after condensation by higher-order structures was termed “heterochromatin”. Condensation of the DNA into heterochromatin makes it inaccessible and protects it from damage – a benefit that is gradually lost with old age [30, 31].

### Histone modifications and heterochromatin decline

Post-translational modifications (PTMs) on histones have multiple effects on gene expression. PTMs regulate chromatin compaction by altering histone-histone or histone-DNA interactions, modulate local gene expression directly and impact the recruitment of effector proteins and transcription factors, thereby controlling gene expression indirectly [31, 32]. There are more than 20 post-translational histone modifications currently described in literature - among these are methylation, acetylation, phosphorylation, ubiquitylation and sumoylation - and their effects are interdependent, making it challenging to study how one specific alteration impacts genetic regulation [33], since it is often a combination of modifications which guides specific regulatory functions [34].

Histone modifications occur in the flexible (and easily accessible) N-terminal histone tails protruding from the nucleosome core or in other histone domains, such as the lateral histone regions or the central globular domains, directly impacting chromatin binding and associated activity [33]. Additionally, histone variants, such as H2A.W and H3.3, gradually replace some of the core histones during aging, promoting chromatin architecture changes, and adding another layer of complexity to an already intricate system [35].

In the last decade, several studies have described how histone modifications work in tandem to regulate genomic expression [36, 37]. The best studied PTMs are acetylation and methylation, which occur primarily on lysine and arginine residues in the histone tails [38, 39]. These residues are likely selected for PTM due to their positive electrostatic charges, which interact with the negatively charged nucleic acid nucleotides forming strong electrostatic bonds [38, 40]. In the case of acetylation, the addition of acetyl groups neutralizes the positive charge of arginine or lysine residues, decreasing the strength with which the modified histones interact with the DNA, resulting in euchromatin formation, increased transcription and increased genomic instability [38, 41]. Nonetheless, a study by Li and colleagues reported that the acetylation of lysine 85 of linker histone 1 resulted in heterochromatin condensation, a clear example of how the same PTM may impact genomic structure differently, depending on which residue is modified and the interactions it establishes [42]. The impact of histone methylation is similarly complex, with the stability of the genome being altered in accordance with the position of the modified residue and its interactions with other PTMs, with some methylated residues favoring genomic transcription in some contexts, while promoting repression in others [43, 44]. For example, methylation of H3K4 (lysine 4 of histone 3) represses transcription in the presence of H3K9 methylation but increases transcription when H3K9 is acetylated [45].

Histone methylation is mediated by lysine or arginine methyltransferases (KMTs and PRMTs, respectively) that add methyl groups to the target histone residues using S-adenosylmethionine (SAM) as a methyl group donor [46]. Demethylation is mediated by histone demethylases (HDMs), that remove methyl groups [41, 47]. Histone acetylation is mediated by histone acetyltransferases (HATs), which add acetyl groups from acetyl-CoA [46]. Deacetylation is mediated by histone deacetylases (HDACs) – which remove those groups [47]. HATs and HDACs have been the target of recent pharmaceutical studies, looking for therapeutic agents that can modulate gene expression in cancer [44, 48] and aging.

Aging is associated with an overall increase in histone acetylation [49]. A group of HDACs known as Sirtuins have been studied as a target for improving healthspan [50]. Sirtuins belong to a family of NAD+ dependent proteins with histone deacetylase function and their activity levels decrease with aging, in tandem with a decrease in NAD+ levels [49, 51]. Members of the Sirtuin family (SIRT), namely SIRT1, SIRT3 and SIRT6, have been shown to promote health and longevity in various model organisms, from yeast to mammals (particularly in situations of caloric restriction and high exercise). As a result, Sirtuins have been considered as potential therapeutic targets to extend healthy aging, either by increasing the availability of NAD+, essential to keeping metabolism at the required rates, or by using pharmacological compounds (such as resveratrol) that directly activate Sirtuins function [49, 52–54].

Several specific PTMs have also been associated with aging including H4K16ac, acetylation of lysine 16 of histone H4 (associated with reduced chromatin condensation), H4K20me2 and H4K20me3, di- and tri-methylation of lysine 20 of histone H4 (which increase chromatin compaction *in vitro*) and H3K9me3, tri-methylation of lysine 9 of histone 3 (which promotes a stronger binding between the DNA and the histone octamer - a modification that is gradually lost with aging) [29, 31, 55].

In addition to changes in acetylation and other PTMs, aging is associated with a global reduction in core histones proteins accompanied by a decline in protein synthesis [17]. The loss of histones implies a decrease in heterochromatin, an observation which led to the “heterochromatin loss model of aging”. This model suggests that there are conformational changes in heterochromatin structure with aging that result in the activation of genes that were previously silenced. These alterations, in turn, cause aging and cellular senescence along with increased genomic instability - further aggravated by chromatin relaxation, leading to age-associated changes in gene expression and increased genome damage [18, 29].

### DNA methylation and aging

DNA methylation (DNAm) is observed in all eukaryotes, from plants and fungi to animals, and it is central to cell differentiation and embryonic development [56]. After replication, a methyl group is added to the fifth carbon of the cytosine base ring (5mC), most commonly in genomic locations that are rich in cytosine-guanine dinucleotides, known as CpG islands [57]. Methylation can also be observed at other nucleotide pairs (adenine-cytosine; thymine-cytosine; cytosine-cytosine), which are known as non-CpG, or CpH sites [39, 58], although it is less frequent than at CpG sites. Non-CpG methylation is particularly common in embryonic stem cells [59] and neurons [60]. DNAm is much more prevalent in heterochromatin than euchromatin [30].

DNAm is mediated by the DNA methyltransferases (DNMTs) 1 and 3, with S-adenosyl methionine (SAM) serving as donor for the methyl group, the same molecule that serves as donor for histone methyltransferases [46, 61]. The function of DNMT1 is to maintain existing methylation sites, by adding methyl groups at CpG sites that are methylated on only one DNA strand, such as after DNA replication. Conversely, DNMT3A, DNMT3B and DNMT3L promote *de novo* DNA methylation [57, 58, 61].

DNMTs activity is balanced by DNA demethylation, which can be either passive or active. In passive or spontaneous demethylation, the cytosine loses its methyl group during replication and is converted to thymine, which is posteriorly exchanged through repair mechanisms to an unmethylated cytosine. Active demethylation is mediated by Ten-Eleven Translocation (TET) proteins. These catalyze the demethylation process through successive oxidations, from 5mC to 5-hydroxymethyl cytosine (5hmC) and afterwards to 5-formylcytosine and 5-carboxylcytosine, which are recognized by Thymine DNA Glycosylase (TDG) and returned to their unmethylated cytosine state by the base excision repair (BER) pathway [57, 62]. There are several proteins involved in this process. Among them, are the protein GADD45A (“Growth Arrest and DNA Damage Protein 45 A”) and the members of the AID/APOBEC (Activation Induced Cytidine Deaminase (AID)/Apolipoprotein B Editing Complex (APOBEC)) protein family. GADD45A recruits TDG in situations of DNA damage [63], and recognizes promoters’ R-loop conformation, leading to TET recruitment and demethylation at CpG islands [64]. In addition, the members of the protein family AID/APOBEC are described as having a role in demethylation through deamination of 5mC cytosine [65], which generates a G-T mismatch that is posteriorly corrected by TDG [66].

In young stages of life, eukaryotic DNA is hypermethylated in repetitive elements of the genome, in intergenic regions and in gene bodies. Hypermethylation promotes genomic stability and limits transcription of these regions of the genome. With aging, there is global hypomethylation of previously methylated regions, leaving these more available to transcription factors and other effectors and consequently promoting transcription of these genomic sites [59]. This hypomethylation is associated with a decrease in DNMT1 expression [67], which likely contributes to impaired maintenance of DNAm patterns associated with aging.

There is also, contradictorily, some evidence of age-associated DNA hypermethylation in genomic regions that in young individuals’ DNA are usually unmethylated. Methylation of these regions is associated with genomic compactation, restricting gene transcription, which may also lead to genomic instability, if genes that should be transcribed find themselves restricted by this event [29, 56, 68].

DNAm alterations are widely described in cancer studies. Hypermethylation of tumor suppressor genes and hypomethylation of oncogenes are both associated with genomic imbalance and dysregulated proliferation of cancer cells [69]. A similar phenomenon is hypothesized to occur in aging, with age-associated genes as the target - in this case, hypomethylation at the promoters of genes regulating senescence may translate into increased expression of these genes and accelerated aging.

Recently, DNAm has been used as a biomarker to measure aging by analyzing methylation status across a large set of CpG sites [70]. This led to the creation of epigenetic clocks (e.g. Horvath’s, Hannum’s, PhenoAge and GrimAge [71, 72], which were designed to predict chronological age based on epigenetic criteria. Subsequently, epigenetic clocks have been used as a proxy for biological age and have been extensively studied for their ability to predict healthspan, disease risk and mortality [73, 74] Epigenetic clocks are reliable predictors of chronological age, but their utility in predicting mortality and healthspan is less clear. Further studies are required to determine whether specific CpG sites can be used to predict healthy longevity [75–78].

### Modulation of aging by non-coding RNAs

Non-coding RNAs (ncRNA) contribute for the control of gene expression as they have key regulatory functions in both physiological and biological processes [79]. As measurable molecules, circulating ncRNAs and particularly microRNAs (miRNAs), are considered promising biomarkers for the study of aging and age-associated processes, as they have been linked to aging (and age-related pathways), cellular senescence and age-associated conditions [80–83].

MiRNAs, the most studied of the ncRNAs, are short molecules of 18 to 25 nucleotides that regulate gene expression by binding to the 3′ untranslated region (3′UTR) of their target genes blocking protein translation or inducing mRNA degradation [84]. There are more than 2800 miRNAs encoded in the human genome, and it is estimated that at least 60% of all human genes are regulated by miRNAs [80, 85, 86]. MiRNAs may also impact other epigenetic events by regulating their associated enzymes. For instance, members of miR-29 family, modulate the expression of DMNTs and TET enzymes in both healthy and pathological conditions, namely brain maturation and cancer [84, 87], leading to the reduction of DNA hypermethylation in tumor suppressor genes [87, 88].

LncRNAs or “long non-coding RNAs” are a diverse class of ncRNAs longer than 200 nucleotides. They can bind to DNA, RNA or proteins and are involved in the post-transcriptional and post-translational regulation of gene expression. It has also been suggested that lncRNAs may interfere with chromatin structure modulation and/or transcription [79, 89].

Both of these ncRNAs have been implicated in senescence and inflammation-related pathways (such as the p23/p21 and the nuclear factor kappa light-chain enhancer of activated B cells (NF-κB)), in the development of neurological disorders (such as Huntington’s and Alzheimer’s disease) [90] and other age associated conditions, such as fibrosis [79], cardiovascular disease [89, 91] and osteoporosis [92]. Thus, ncRNAs are increasingly being targeted for anti-aging therapies [93].

ncRNAs influencing aging include miR-28e3p and miR-126, whose levels are reduced in diabetes mellitus type 2 [94], and miR-146a, that inhibits NF-κB pro-inflammatory activity and shows reduced expression levels associated with aging [6]. Another ncRNA, miR-21, was demonstrated to modulate inflammation and the NF-κB pathway and shows altered levels in cardiovascular diseases and osteoporosis [92, 95]. Another ncRNA that has been linked to aging is nc886 (or pre-miR-886), which was shown to impact senescence by reducing the expression of senescence biomarkers, such as p16^INK4A^ and Cyclin Kinase Inhibitor p21 (p21^Waf1/Cip1^), and decreasing reactive oxygen species (ROS) levels in fibroblast cell models. This molecule is under investigation as a potential target for anti-aging therapy [96].

In short, the aging process leads to alterations in non-coding RNA levels, which in turn modify the expression levels of age-related genes and increases age-related genomic instability.

## Application of nutritional and lifestyle strategies to epigenetically modulate healthspan

Studies have shown that from an economic point of view is more advantageous for society to promote even a slight increase in healthspan, rather than investing in disease-specific adaptions that cater to an aged population [16, 97]. Henceforth, it is important to identify biomarkers and develop biological clocks that can assess the value of novel strategies that may potentially overcome or delay conditions leading to poor health amongst the elderly [98–100].

Over the years, there have been many theories about how to extend healthy lifespan: from eating superfoods and following fad diets, to adopting specific behavioral habits – making it difficult to distinguish what is myth from what is backed up by scientific evidence. While there is broad scientific consensus that daily habits impact aging, there is no magic solution, much less a “one size fits all” approach to achieving a long and healthy life. Nonetheless, some practices have shown promising results in scientific studies [97].

One lifestyle factor that has an impact on healthspan is literacy [101]. Intriguingly, low levels of literacy are associated with epigenetic changes, similar to the effect of alcohol consumption [102, 103]. Other lifestyle factors that impact epigenetic age acceleration include living environment, stress levels [104, 105], the complexity of an individual’s social network and their economic power [99, 102]. Lack of sleep [106, 107], coffee and tea consumption [108, 109], and smoking [110] are also associated with similar epigenetic age acceleration.

In the following sections, we examine the evidence for the impact of lifestyle factors on healthy aging and present suggestions for ways to promote healthy aging through simple changes regarding nutrition and physical activity.

### The impact of nutrigenomics on healthspan

Nutrigenomics is the study of nutrients and diet, and of their influence on the epigenome. It aims to describe, characterize, and understand the mechanisms by which our dietary intake influences gene expression. The field of nutrigenomics has emerged with the adoption of better sequencing technologies, such as next-generation sequencing (NGS), and the development of precise techniques for whole-genome chromatin analysis, namely HiC and ATAC-seq (which stand for “High-throughput Chromosome Conformation Capture technique” and “Assay for Transposase-Accessible Chromatin with high-throughput sequencing”, respectively) [111–113]. These technologies have enabled scientists to study the impact of nutrition on gene expression both in the short and long term [111, 112].

Popular wisdom suggests that there are multiple diets and foods that can promote healthy aging. However, few of these have been shown to positively impact aging and aging associated pathologies. Evidence does exist for the Mediterranean and the Okinawan diets, both of which are associated with lower levels of inflammation and oxidative stress, reduced incidence of cancer, as well as a decreased risk of cardiovascular disease [114–116].

Both the Mediterranean and Okinawan diets include low glycemic index foods and favor the consumption of seasonal local foods - particularly fruits, vegetables, and nuts – along with moderate intake of animal products, olive oil, spices and red wine. They ensure a proper intake of valuable nutrients (see Table 1), while preventing overeating [115, 117, 118]. In epigenetic studies, both diets have demonstrated anti-aging benefits in human subjects (Table 1) [114], and they have also been shown to reduce epigenetic age, according to the DNAGrimAge epigenetic clock [72, 102].

**Table 1:** Food-induced epigenetic alterations.

**Compound**	**Foods**	**Overall impact**	**Epigenetics impact**	**References**
Betalains (Indicaxanthin)	Red Beetroot; Cactus Pear (*Opuntia Ficus Indica*)	Antioxidant, Anti-inflammatory activity, Anti-carcinogenic	**DNAm modulator**: increases the expression of genes involved in DNA demethylation of tumor suppressor gene promoters	[216, 217]
Catechin/Epicatechin (EGCG)	Green Tea, Cocoa, Apple	Antioxidant, Neuroprotective, Anti-inflammatory activity, Anti-carcinogenic	**MiRNA modulator**: increases expression of oncosuppressor miRNAs (i.e., miR-29)	[88, 133, 218]
Curcumin (diferuloyl-methane)	Turmeric	Anti-inflammatory activity	**DNAm modulator**: DNMTs’ regulating functions through hypomethylation of oncosuppressor genes; **Histone modifications**: regulates HATs and HDACs; **MiRNA modulator**: increases expression of oncosuppressor miRNAs	[122, 219, 220]
Hydroxytyrosol Oleic acid	Olive oil	Antioxidant, Cholesterol and Low-Density Lipoprotein (LDL) reductor	**MiRNA modulator**: increases expression of oncosuppressor miRNAs, as well as miRNAs associated with fatty acid biosynthesis (let-7e-5p) and age-associated signalling (miR-17-5p)	[117, 126, 127]
Lycopene	Tomato	Antioxidant, Anti-inflammatory activity	**DNAm modulator**: high lycopene levels coincided with T-cell signalling protein hypermethylation and altered T-cell signalling pathway in “head and neck cancer survivors”	[121, 221]
Omega-3 fatty acid	Fish oil (i.e., Sardine, Salmon), Nuts	Anti-inflammatory activity, Antioxidant	**Histone modifications**: suppresses HDACs, promoting gene transduction; **DNAm modulator**: alters TET1 expression; **MiRNA modulator**: upregulates hsa-miR-551a (tumor suppressor miRNA)	[222–224]
Quercetin	Tomato, Onion, Capers	Anti-inflammatory activity, Anti-carcinogenic, Neuroprotective	**MiRNA modulator**: increases expression of oncosuppressor miRNAs	[131, 225, 226]
Resveratrol	Grapes, Nuts, Berries, Red Wine	Anti-inflammatory activity, Antioxidant, Vasoprotective properties; Neuroprotective	**Histone modifications**: inhibits HATs and HDACs; **DNAm modulator**: regulates DNMTs; **MiRNA modulator:** upregulates the tumor suppressor miR-let7A	[53, 182]
Sulforaphane/isothiocyanates	Broccoli, Cabbage, Kale	Anti-carcinogenic, Anti-inflammatory activity, Antioxidant, Proteostasis promoter	**Histone modifications**: inhibits HDACs; **DNAm modulator**: regulates DNMTs, by decreasing DNMTs’ expression and promoting the activation of tumor suppressor genes	[227–229]
Sulfur-containing compounds, i.e., diallyl trisulfide (DATS) and S-allylcysteine (SAC)	Garlic	Anti-carcinogenic	**DNAm modulator**: DNMTs’ regulating functions, suppressing tumor proliferation; **Histone modifications**: reduces HATs and HDACs activity	[230, 231]

Organization by alphabetical order of compounds. For each compound there exists at least one study performed in human subject.

“Superfoods”, such as algae [119, 120], curcumin [121, 122], kale [123, 124], and olive oil [125–127] as well as particular nutrients and/or food components, such as anti-oxidants [53, 128, 129], vitamins [113, 130] and polyphenols [131–133] have also been studied to determine their impact on epigenetics. In Table 1, we present food-associated epigenetic alterations reported in the scientific literature with evidence from human studies.

### Caloric restriction

Caloric restriction (CR), reducing an individuals’ caloric intake by 10% to 40% without compromising nutritional value, has been shown to have a significant and sustained effects on health and lifespan in several model organisms, from yeasts to mice, as well as in humans [134–137]. In communities with extended longevity, particularly the Okinawa region of Japan, there is evidence of reduced calorie consumption, which several researchers have suggested is a key reason for increased healthspan [1, 138–140]. In addition, there are reports of individuals practicing CR who have achieved remarkable healthspan [141, 142]. A concept associated with caloric restriction is hormesis, which may be defined as the adaptative response of an organism to its exposure to chemical compounds or environmental factors. Dietary restriction is considered an environmental factor on hormetic studies [143].

At a cellular and molecular level, the benefits of CR include an increase in DNA repair - by promoting the maintenance of BER activity [144]-, delayed neurodegeneration in the central nervous system, improvement in glucose metabolism [145], a reduction in the incidence of diabetes and cancer, and a reduction in epigenetic aging-associated events, namely age-associated global hypomethylation [1]. CR is also closely related with autophagy. This is in large cause due to nutrient depletion, which leads to a reduction of intracellular acetyl coenzyme A (AcCoA) and consequentially to protein deacetylation [146].

On a physiological level, CR exerts its influence through nutrient-sensing pathways [147] by generating a cascade that begins with a reduction in blood glucose levels, leading to an increase in insulin sensitivity [148] and to a reduction in insulin/insulin-like signalling and its’ associated pathways such as IGF-1 pathway [149]. These alterations decrease cell growth and proliferation and promote cell maintenance through repair mechanisms [150]. Furthermore, the reduced availability of nutrients also inhibits the serine/threonine protein kinase mechanistic Target Of Rapamycin (mTOR) pathway and FOXO proteins, particularly FOXO3 [151]. FOXO3 and SIRT1 are phosphorylated by AMPK, whose activation leads to a decrease in protein synthesis and to the referred increase of autophagy at the cellular level [151, 152] as well as ketogenesis and fatty-acid oxidation in the liver [141].

One of the results of CR is weight loss, that diminishes the risk of age-associated diseases, namely cardiovascular, as well as an increase in lifespan [1, 139]. The CALERIE 2 study, which studied the impact of CR on longevity in humans, recruited over 200 healthy participants who maintained 25% CR for two years. In this study, CR led to improvements in multiple dimensions including quality of life, sleep and sexual function of the participants [153].

At the epigenetic level, CR has a significant impact on epigenetic events associated with aging. CR delays DNAm age-related alterations [136, 145], such as the increase in DNMT3a immunoreactivity [154], reduces histone modifications, partly through Sirtuin activation [155], and alters microRNA activity [156], namely miR-125 whose target gene - *chinmo* - impacts fat metabolism and longevity [157]. Interestingly, a study by Maegawa and colleagues, in 2017, reported a significant decrease in age-related methylation progression in Rhesus monkeys who maintained 30% to 40% CR during approximately 10 years. The monkeys also showed epigenetic age deceleration relative to their chronological age [158]. The benefits of CR are further supported by a study from Pifferi and team in 2018 that demonstrated a 50% increase in the lifespan of the grey mouse lemur in response to 30% CR. There was also a significant reduction in age-associated diseases, compared to a control group with a normal diet [159].

Notwithstanding these advantages, CR is not easy to adopt, which led to the development of intermittent fasting approaches in the hopes of reaping some of the benefits of CR [160]. These range from alternating fasting with normal feeding on different days of the week [139, 161] to restricting the time of the day individual eats (time-restricted feeding) [160, 162]. However, the health benefits of these strategies are not as clear as the benefits provided by classic caloric restriction.

The benefits of CR need to be balanced against potential negative effects [141]. A study of grey mouse lemurs reported grey matter reduction in the group that underwent 30% CR [159]. Extreme CR may also result in significant weight loss and decreased body fat, which can lead to health complications such as bone density disorders (osteoporosis) and compromised healing [163], infertility, and increased cognitive impairment and weight gain in offspring [164]. There is also the risk of eating disorders, such as anorexia and bulimia [165].

For these reasons, there is great interest in pharmaceutical agents that provide the beneficial effects of CR without the need to undergo a calorie-restricted diet [166]. Several drugs are already in clinical trials [167], while other compounds are being commercialized by emerging companies [168]. One class of potential CR mimics is the NAD+ precursors [169] including nicotinamide mononucleotide (NMN) [170, 171] and nicotinamide riboside (NR) [172]. These molecules have been shown to have a preventive effect on age-associated conditions. FOXO3 transcription factor is also emerging as a therapeutic-target in age-related diseases and healthspan focused studies, as it has been associated with longevity in human and animal model studies [173–175]. Since proteins from this family group are mainly regulated through PTMs (namely by HATs and HDACs), they become particularly desirable targets to drug-based treatments [173, 174]. Moreover, in the USA it was established a consortium named “Interventions Testing Program (ITP), where drugs are tested to assess their effects in mice’ lifespan [24, 176].

Some polyphenol treatments have also demonstrated to mimic CR effects by interacting with longevity associated signalling pathways and molecules [50, 52, 138, 150, 177]. Among these we can find resveratrol (presented in Table 1) [146], metformin (an anti-diabetic drug used in type 2 diabetes mellitus to control high blood sugar) [178, 179] and rapamycin [180] that demonstrated not only to have anti-inflammatory and anti-oxidant activities, and to promote autophagy, but also to induce positive aging-associated epigenetic alterations:

- In an extensive revision work performed by Pyo et al., the mechanisms through which resveratrol impacts aging in humans and in different animal models were thoroughly described. It was considered that the main mechanisms involved were the activation of proteins from Sirtuin family (such as SIR2 and SIRT1, which have deacetylase activity) and peroxisome-proliferator-activated receptor-g coactivator-1α (PGC-1α), and regulation of the AMPK pathway [53, 181, 182]. Furthermore, studies with human old aged subjects demonstrated that resveratrol supplementation decreased inflammation markers such as Interleukin (i.e., IL-6) and Tumor Necrosis Factor-α (TNF-α), and reversed histone PTM markers of aging, particularly through modulation of acetylation [182–184]. In addition, a study performed by Jimenez et al. showed resveratrol is able to induce nuclear translocation of FOXO3 and to activate this protein independently of phosphoinositide 3-kinase (PI3K)/AKT signalling [173].
- Studies developed using metformin - both as a therapeutic intervention on aging and age-associated diseases as well as on the hallmarks of aging themselves - further demonstrated the potential of this compound, through modulating IGF-1 and AMPK activation and mTOR pathways [185, 186]. It was demonstrated that this drug impacts the epigenetic events described above by altering the activity of HATs, HDACs and DNMTs enzymes, which are phosphorylated by AMPK [187]. Moreover, a study by Cuyas and colleagues computationally predicted metformin’ targets, including several epigenetic modifiers, among which KDM6A/UTX, a member of the H3K27me3-specific demethylase subfamily [188].
- Rapamycin is a drug able to mimic CR by compromising a cell capacity to sense the nutrients in its environment [150]. It targets the mTOR pathway, by binding to FK-506 binding protein 12 (FKBP12) to establish a complex of three different molecules, which inhibits the mTORC1 subunit. This mechanism showed advantages when applied to disease models, in both animal models and humans [150, 180, 189, 190], as well as lifespan, by increasing autophagy rates. Additionally, the impact of rapamycin on epigenetics has also been demonstrated in several studies. It was shown that it not only maintains young markers of DNAm in mice [191], but also that it promotes the recovery of histone methylation markers which are lost with old age in mice brain tissues [189].

Henceforth, it is possible to observe the closeness of the mechanisms described as associated to CR and to the compounds presented, further reiterating the similarities between them. Furthermore, this also suggests the potential of these strategies as being both complementary and extensions of each other.

### The impact of physical exercise on epigenetics

Another strategy to achieve extended healthspan is physical exercise. Physical activity may be defined as “any movement executed by skeletal muscles, that requires energy expenditure” [48, 192]. Its opposite, sedentary behavior, is known to increase age-associated conditions including cardiovascular conditions, metabolic syndrome [193], incontinence [194], cancer, cognitive decline, and neurodegenerative disorders [4, 195–197]. Studies have demonstrated that even a slight increase in physical activity positively impacts healthspan and reduces frailty, as well as having neuroprotective effects and improving cognition [198, 199]. It also reduces age-associated conditions and reduces inflammaging (a generalized increase in circulating pro-inflammatory cytokines, such as Interleukin-6 (IL-6) and Tumor necrosis factor α (TNF-α), that is associated with old age) and immunosenescence [48, 200].

Contrasting with the topics of nutrition and caloric restriction, the cellular and molecular mechanisms underlying the health benefits of physical activity in humans remain poorly understood [201, 202]. In a study performed by Hoshi et al., 145 bioactive lipids (cellular signalling molecules which are strongly related with immunity regulation, inflammation and homeostasis) were associated with higher levels of physical activity, 12 of which were shown to be inversely correlated with cardiovascular disease events [202]. Moreover, a genome-wide association study by Wang et al. screened genomic data from approximately 704 000 individuals with differing physical activity habits. They targeted 99 loci, where 104 independent association signals were identified, among which p.Glu635Ala, a missense variant rs2229456 of *ACTN3*. This variant was further explored using molecular dynamics simulations, and was associated with increased “moderate-to-vigorous intensity physical activity during leisure time” habits, likely “by reducing susceptibility to exercise-induced muscle damage” [203]. O’Reilley and colleagues reviewed how physical activity relates with mitochondrial remodelling and how these, in turn, influence neurodegeneration. They highlighted how mitochondrial function strongly benefits from aerobic exercise in some tissues, and that this may positively impact the brain by delaying the age-associated increase of oxidative stress that leads to mitochondrial decline [204]. Furthermore, a review by Reddy et al. explores the “exercise responsome” and several molecules (exerkines) which are released during physical activity (such as Cathepsin B, Adiponectin, Osteocalcin and FGF21) [205, 206]. In this same study, exercise mimetic-drugs are studied and it is proposed that AMPK-SIRT1-PGC1α-BDNF pathway is the main mediator of the “cognitive benefits associated with exercise” [205].

On an epigenetic level, regular exercise leads to slower progression of the DNA methylation alterations associated with age [207–209], and to beneficial changes in miRNAs that regulate inflammation levels [48]. The epigenetic effects of physical exercise on human subjects reported in the literature are presented in Table 2. These studies demonstrate the many positive impacts of physical exercise on health status, by reducing age-associated and disease-associated epigenetic changes.

**Table 2:** Exercise-associated epigenetic alterations.

**Exercise**	Individuals tested^*^	**Intensity/Frequency during/in study**	**Epigenetic impact**	**References**
Aerobic and resistance training (jogging, cycling, swimming, and others)	**Healthy** (male marathon runners)	Immediately after and 24h post marathon;	**MiRNA modulator:** changes in circulatory miRNA levels after racing	[232]
	**Healthy (**male cyclists)	Before and after the supplementation in EVOO ^**^ - alongside cycling - 4 weeks	**DNAm modulator:** levels of DNMT3A and DNMT3B mRNA expression decreased following exercise	[233]
	**Non-Healthy** (obese)	3 months study, 2 times per week, 90 minutes session;	**MiRNA modulator:** i.e., miR-146a-5p levels were significantly decreased after intervention - positive impact in inflammation	[234]
	**Non-Healthy** (hypertensive)	3 months study, 4 times per week, 40 minutes session	**DNAm modulator**: repetitive elements methylation (i.e., ALU), potential role in reducing systemic blood pressure	[208]
	**Non-Healthy** (colorectal cancer survivors)	6 weeks - resistance exercise training - samples collected before and after intervention	**DNAm modulator:** changes in promoters of biologically related genes of processes such as immune response and disease; reduced methylation of disease preventive genes	[235]
	**Aged** (women; 68 ± 7.5 years old)	4 study groups: resistance training, water aerobics, water aerobics and resistance exercise, and control group (non-practitioners) individuals, with 3+ months of practice, 2/3 times per week	**DNAm modulator:** increased global and gene specific (Interleukin-17 (IL-17A) and Interferon gamma (IFN-γ)) DNA methylation - positively impacting inflammation	[236]
	**Aged** (70–75 years old)	12 weeks, low frequency, moderate intensity, explosive-type resistance training	**DNAm modulator:** reduction of global DNAm levels, together with better leukocyte telomere length maintenance	[237]
Relaxation and stress targeted exercises (Tai Chi, Meditation, Yoga)	**Healthy** (women – 45 to 88 years old)	Tai Chi practitioners for 3+ years, for at least 1 hour per week)	**DNAm modulator:** decreased age-associated DNAm levels - trend more evident in older subjects (>55y)	[207]
	**Healthy** (individuals younger than 52 years old and individuals aged 52 or older)	Meditation practitioners for 3+ years, for at least 30 minutes daily	**DNAm modulator:** Intrinsic Epigenetic Age Acceleration (IEAA) similar in practitioners younger and older than 52 years old. In the control group there were significative differences between age groups, with higher IEAA in the ≥ 52 group.	[211]
	**Healthy** (30 to 65 years old)	Yoga and Meditation Based Lifestyle Intervention (YMLI) 12 weeks, 5 days per week, 90 minutes per day	**Genomic stability:** DNA damage and genomic instability reduction. Cellular aging biomarkers improvement: increased balance of inflammation and cellular oxidative stress levels	[238]
	**Non-Healthy** (infertile males)	21 days, daily Yoga practice – 1 hour per day Collection of samples before and after intervention	**DNAm modulator:** over 400 DNAm changes observed in fertility-associated gene promoters, together with improved sperm parameters	[239]
	**Aged** (community dwelling 60 + years old)	1 month, daily Tai Chi practice - 1 hour per day	**DNAm modulator:** Brain-derived neurotrophic factor (BDNF) promoter demethylation - marker of depression recovery: marked results in depression symptoms improvement	[240]

^*^The individual tested/participants were discriminated in their respective studies in accordance to medical conditions, gender and age. ^**^EVOO stands for Extra Virgin Olive Oil.

The World Health Organization has promoted the philosophy that “some (exercise) is better than nothing”, even if it is just part of an individual’s daily routine, associated with everyday tasks [192]. However, studies have demonstrated that consistent physical activity of variable intensity has more impact on biological aging than exercise done in a purely occupational context [210], and research also described that exercises targeting relaxation - such as yoga and meditation -, show a decrease of Intrinsic Epigenetic Age Acceleration (IEAA) [211]. Moreover, the epigenetic benefits that appear with exercise are severely reduced or disappear shortly after physical activity interruption [212], so consistency in physical activity is likely to be important.

It has been shown that DNAm events are influenced by physical exercise and physical fitness. There are emerging DNAm clocks which have presented promising results in estimating healthy aging and its’ progression. One of those is DNAmFitAge, which demonstrated to distinguish between individuals who are highly fit from individuals with low to medium fitness, while also relating verbal short-term memory to decelerated aging [213]. Another DNAm clock is the DNAm GrimAge clock, which shows predictive capacity of lifespan and healthspan and a strong relationship with lifestyle factors, including physical activity [72, 214]. Moreover, several other epigenetic clocks, including the Hannun’s, Horvath’s and PhenoAge [215] associated aging markers with physical activity and disease.

## CONCLUSION AND FUTURE PERSPECTIVES

**Figure 2:**
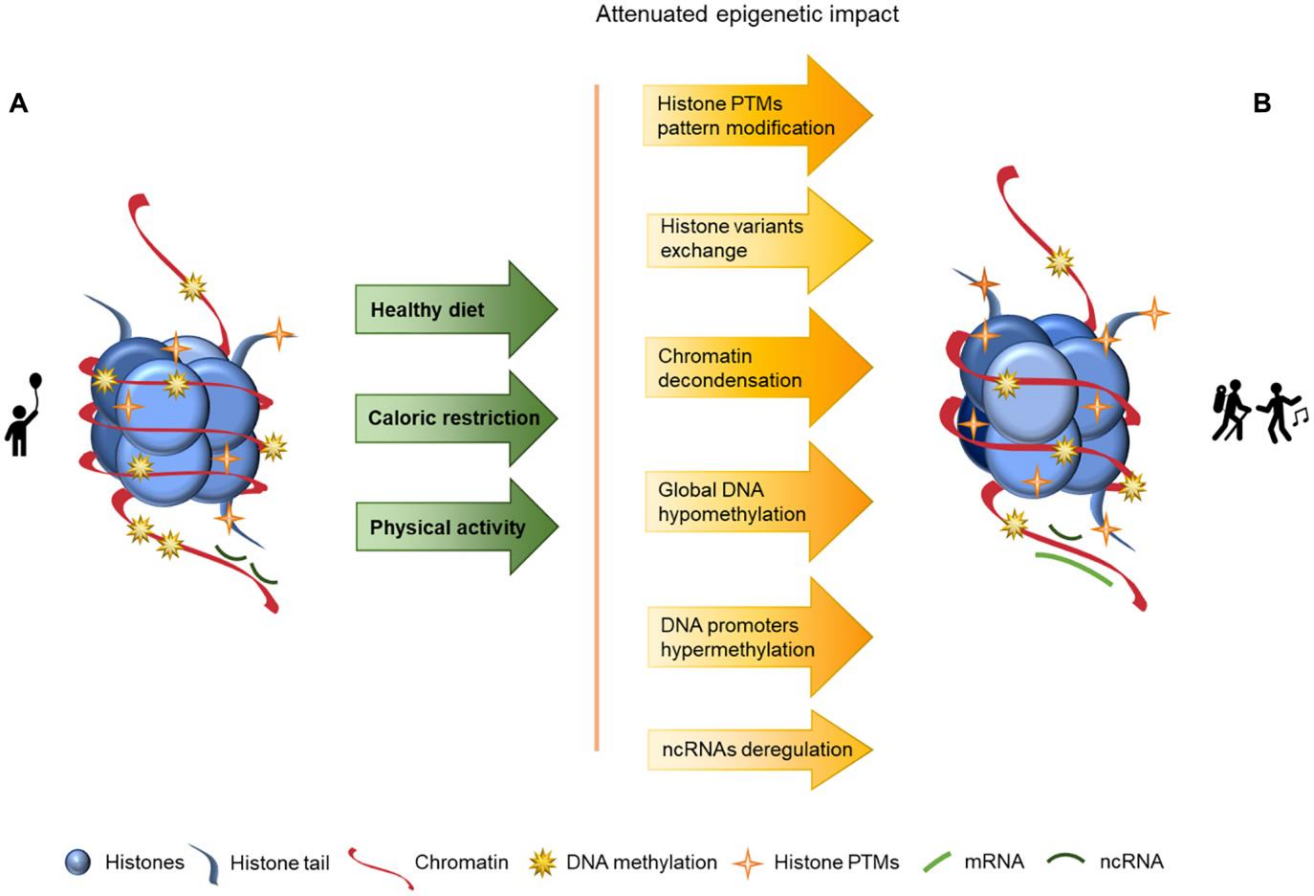
**Representation of age-associated epigenetic changes after following a healthy lifestyle.** (**A**) Representation of a young individual chromatin, with tight chromatin compactation, high levels of DNA methylation, decreased histone PTMs (particularly acetylation), canonical histones and balanced non-coding RNA regulation; (**B**) Representation of a healthy old aged individual chromatin, we may observe a looser chromatin structure and lower levels of DNA methylation than in A, and higher levels of histones PTMs (acetylation) than in A. There are also different histone variants presence (in exchange of the canonical histones) and an increase of ncRNA imbalance, which is reflected in an overall increase of chromatin instability. The alterations between the structures presented in A and B are represented by arrows. The arrows in green present the causes (here presented as a mindful lifestyle - achieved through a healthy diet, caloric restriction and physical activity) whose effects are reflected by the arrows in yellow/orange, with the lighter colors representing less marked epigenetic alterations.

Aging is a complex and natural process in every living organism’s life cycle and it manifests in many ways, including epigenetics. In this article we explore aging and its associated epigenetic changes as well as how these changes may be delayed or reversed through nutrition, caloric restriction and sustained physical activity, as schematized in Figure 2. In this Figure we may observe a looser chromatin structure in the component B than in the component A, as well as lower levels of DNA methylation and increased histone PTMs, but nonetheless these values are closer to the component A than to the component B presented in Figure 1, where lifestyle strategies to promote a healthy aging are not considered, and consequentially imply an overall increase of chromatin instability.

It is important to note, however, that epigenetic events are dynamic and interdependent. Moreover, the relationship between age-associated epigenetic changes and healthspan is not always well-established and one epigenetic modification may lead to folding or unfolding of the chromatin structure depending on other nearby modifications. Future epigenomic studies must adopt a genome-wide perspective, rather than a targeted approach, and should adopt a three-dimensional perspective to give deeper insight into their impact on chromatin structure. Furthermore, it would be relevant to develop strategies that could enable the differentiation of standard and healthy older aging from age-associated pathologies themselves.

The aging global population is placing ever-growing demands on social and health infrastructure. Strategies to promote health aging are important to maximize quality of life for the elderly and minimize pressure on health care systems. The simplest “treatments” are lifestyle changes including healthy eating and regular physical exercise. Pharmacological or biological treatments are also in development, and may, in the future, help reduce the risks associated with unhealthy behaviors, such as smoking and drug abuse. Understanding the relationship between biological aging and healthspan is critical to assessing the value of these interventions and identifying new therapies that can promote healthy aging.
